# Postoperative ^68^Ga‐DOTATATE positron emission tomography has a low yield in incidental appendiceal neuroendocrine tumours

**DOI:** 10.1111/ans.19216

**Published:** 2024-08-23

**Authors:** Emilie Pauvert, George Larcos

**Affiliations:** ^1^ Sydney Medical School University of Sydney Sydney Australia; ^2^ Royal Darwin Hospital Tiwi Northwest Territories Australia; ^3^ Department of Nuclear Medicine and Ultrasound Westmead Hospital Westmead New South Wales Australia

**Keywords:** appendix, neuroendocrine tumours, PET imaging

## Abstract

**Introduction:**

Rarely, appendiceal neuroendocrine tumours (NET) are an incidental finding when an appendicectomy is undertaken for suspected appendicitis. The role of further imaging in this setting is poorly defined. Positron emission tomography (PET) using ^68^Ga‐DOTATATE is requested to evaluate post‐surgical status, however, there is little evidence to guide how it should be employed. The aims of this project are to: (i) characterize ^68^Ga‐DOTATATE PET findings in patients with incidental appendiceal NETs and (ii) discuss how these data might inform post‐surgical imaging with PET.

**Methods:**

We reviewed 47 PET scans in 30 patients, undertaken from 2009 to 2018. Scintigraphic findings, histopathological characteristics of the initial appendiceal lesion and medical records were reviewed.

**Results:**

Most patients (*n* = 15) had small (<10 mm) appendiceal NETs with low grade (Ki67 < 2%) features. Eight patients had tumours between 10 and 20 mm, and seven had tumours >20 mm. Goblet cell features were identified in two patients. Three positive PET scans were reported in one patient with an index tumour measuring 40 mm and Ki67 < 2%. The remaining 29 patients had 44 negative scans. Clinical outcome data were available in 27 patients (mean follow‐up time 57 months; range 6–123 months). There was no evidence of recurrent neuroendocrine disease at the time of the last follow‐up.

**Conclusion:**

These data indicate that in most cases, post‐surgical ^68^Ga‐DOTATATE PET is negative in patients with incidentally detected appendiceal NETs. Clinical outcome data suggest that ^68^Ga‐DOTATATE PET should be reserved for patients with large tumours (>20 mm) or those displaying goblet cell features.

## Graphical abstract

Appendiceal neuroendocrine tumours are rarely detected after appendicectomies for appendicitis. Our data show that postoperative imaging with DOTATATE PET is often negative and thus, its use should be reserved for patients with high‐risk features.

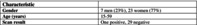

## Introduction

Appendiceal neuroendocrine tumours (NETs) are the most commonly detected neoplasm of the appendix^1^, ^2^ and the third most common neuroendocrine tumour (NET) of the gastrointestinal tract (GIT).^3^ Although the incidence is low (up to five cases per 100 000 people),^1^ an increase has been observed within the past decade.^4^, ^5^ Appendiceal NETs are most frequently detected following appendicectomy for appendicitis and have been demonstrated in up to 2.3% of appendicectomy specimens.^1^, ^3^ These tumours have a slight preponderance towards females and are typically identified in a younger cohort than other GIT NETs.^2^, ^6^

Although most appendiceal NET have a high 5‐year survival rate,^2^, ^7^, ^8^ postoperative management can differ based on various histopathologic findings, most notably lesion size, location and grade.^9^, ^10^ For example, the majority of tumours identified are ≤10 mm,^11^ and appendicectomy alone is generally considered curative, as 5‐year survival data are favourable.^10^ In contrast, tumours >20 mm carry a higher risk of metastatic disease, with up to 40% of these tumours having metastases at diagnosis.^2^, ^3^ In this instance, further surgical intervention, most commonly a right hemicolectomy, is recommended.^2^, ^3^, ^12^, ^13^ Although there is consensus on small and large tumours, there is less clarity on appendiceal NETS between 10 and 20 mm; informing management strategy in this cohort is important as these are not insubstantial in proportion and up to 10% can have metastases at presentation.^2^

The 2023 European Neuroendocrine Tumour Society (ENETS) consensus statements offer imaging guidance on lesions less than 10 mm and those more than 20 mm. In contrast, there is uncertainty about the need for postoperative imaging in patients with appendiceal NETs measuring between 10 and 20 mm.^1^, ^2^, ^3^, ^4^, ^12^ Computed tomography and magnetic resonance are options, but radionuclide imaging using a somatostatin receptor agent, such as ^68^Ga‐DOTATATE (Gallium‐68 Dodecanetetra‐acetic acid conjugated to tyrosine‐3‐octreotate) with positron emission tomography (PET), is of appeal because it affords the opportunity for whole body imaging, as well as offering excellent sensitivity and specificity for well‐differentiated NETs.^14^ Despite a paucity of data on the role of PET in these patients, we have anecdotally noted referrals for postoperative ^68^Ga‐DOTATATE PET in this context. However, there is potential for unnecessary imaging, patient anxiety, complications from supplementary tests, exposure to ionizing radiation and preventable health care costs.

We hypothesize that post‐surgical ^68^Ga‐DOTATATE PET is not routinely indicated in most incidentally discovered appendiceal NETs, especially those smaller than 20 mm. Accordingly, the purposes of this retrospective research are to: firstly, characterize demographic, histopathologic and scintigraphic findings in patients with resected appendiceal NETs; and secondly, to help inform the role of ^68^Ga‐DOTATATE PET in the postoperative management of incidentally detected appendiceal NETs.

## Methods

We reviewed all patients who were referred for a ^68^Ga‐DOTATATE PET scan for the indication of an appendiceal NET between 2009 and 2018. Patients with previously known appendiceal NETs were excluded. Patients underwent standard imaging with an intravenous administration of ~120 MBq of radiopharmaceutical, followed by a 45–60 min uptake period. Imaging data were acquired on a Siemens mCT PET/CT scanner. Typically, patients were scanned for 20–30 min, covering the vertex to proximal femora. Images were reported by an experienced nuclear medicine physician with access to all relevant clinical, imaging and histopathology data. Scans were considered positive if there was evidence of residual disease in the surgical bed, within regional nodes or at distant sites.

Patient demographics, clinical outcome and histopathological data, including size, location within appendix, presence of meso‐appendiceal invasion, Ki67 index, World Health Organisation (WHO) grade and presence of multicentricity, were also determined on review of electronic medical records and correspondence. The study was approved by the institutional research and ethics committee (HREC‐4471).

## Results

Thirty‐eight patients underwent ^68^Ga‐DOTATATE PET between 2009 and 2018 for an appendiceal NET. Thirty of these were incidentally detected following an appendicectomy for appendicitis. Forty‐seven ^68^Ga‐DOTATATE PET scans were performed in these 30 patients. Twenty‐seven patients had available follow‐up data (mean follow‐up period = 57 months [range, 6–123 months]).

Most patients were female (77%) and the median age at diagnosis was 28 years (range, 15–59 years). Most appendiceal NETS were ≤ 20 mm, with the tip of the appendix being the most common location. The presence of meso‐appendiceal invasion was principally seen in tumours >20 mm.

Most specimens had a low Ki67 index (mean = 5%; range = 0–60%) and 21 were Grade 1. Two patients had tumours with goblet cell (GC) features (Ki67% = 23 and 60, respectively) and these were the only Grade 3 tumours observed in our study.

Twenty‐nine of the 30 patients had negative PET scans (Tables 1 and 2). There were three positive scans in one patient, a 50‐year‐old man who underwent an appendicectomy for suspected appendicitis and was found to have a 40 mm NET at the tip of his appendix; this tumour demonstrated no signs of local invasion and had a low Ki67 (<1%) on histopathology. His first PET scan (performed 1 month post‐initial surgery) demonstrated uptake within the terminal ileum (consistent with residual disease), which was managed surgically. His second PET (performed 8 months after initial surgery) demonstrated para‐aortic lymph node involvement, which was managed with chemotherapy. His third scan (performed 26 months after initial surgery) demonstrated a liver metastasis, which was managed surgically. This man was subsequently disease free at 83 months following initial presentation. All other patients were well at the last recorded clinical visit and none had evidence of residual or recurrent disease.

**Table 1:** Clinical and scintigraphic characteristics of cohort

Characteristic	
Gender	7 men (23%), 23 women (77%)
Age (years)	15–59
Scan result	One positive, 29 negative

**Table 2:** Histopathology characteristics

Size	<10 mm	10–20 mm	>20 mm
	15 (50%)	8 (27%)	7 (23%)
Grade 1	12	4	5
Grade 2	3	2	2
Grade 3	0	2* (both with goblet cell features)	0
Location			
Tip	12	3	3
Middle	1	2	2
Base	0	3	1
Multicentric	2	0	1
Meso‐appendiceal invasion	4	1	6
Right hemicolectomy performed	3	6	7

Among the other patients, 15 of 29 underwent a right hemicolectomy (three in patients with tumours <10 mm, six with tumours 10–20 mm, and seven of those with tumours >20 mm). We tabulated scintigraphic and histologic data in 23 subjects who underwent a right hemicolectomy after the diagnosis of appendiceal NET had been made. Although eight of these subjects did not have clinical outcome data (and thus were excluded from the main cohort), their findings have nevertheless been included (Table 3) to portray more detail regarding histology and scan results.

**Table 3:** Clinical, scintigraphic and histologic data in right hemicolectomy patients

Gender	Scan result	Multicentricity	Tumour size(s) (mm)	Grade	Ki67 (%)	TNM classification	Perineural, lymphovascular nodal or meso‐appendiceal involvement
M	Negative	Yes	1.5–9	1	<2	T3 N0 M0	No
M	Positive	No	5	1	1	T1 N1 M1c	No
F	Negative	No	6	1	<2	T3 N0 M0	No
M	Negative	No	8	1	1–2	T1 N0 M0	No
F	Negative	No	8	1	<1	T3 NX M0	Yes
F	Negative	No	10	2	<5	T3 N0 M0	No
F	Negative	No	14	1	1–2	T3 NX M0	Yes
F	Negative	No	14	2	5.8	T3 N0 M0	Yes
F	Negative	No	15	1	<1	T1 N0 M0	No
F	Negative	No	18	1	<1	T3 NX M0	Yes
F	Negative	No	20	3	>60	T2 NX M0	Yes
M	Negative	No	20	3	23	T3 N0 M0	No†
F	Negative	No	22	1	1.4	T3 NX M0	Yes
F	Negative	No	22	1	<2	T3 N0 M0	Yes
F	Negative	No	22	2	3	T3 NX M0	Yes
M	Negative	No	25	1	<2	T2 N1 M0	Yes
F	Negative	No	25	1	<1	T3 NX M0	Yes
F	Negative	No	25	2	>2	T3 NX M0	Yes
F	Negative	No	35	1	<1	T3 N0 M0	Yes
F	Negative	No	Diffuse	2	5	T3 N0 M0	Yes†
M	Negative	No	Diffuse	3	80	T3 M1 N1a	Yes†
M	Negative	No	UA	2	10	T3 N1 M0	Yes†
M	Negative	UA	UA	UA	UA	T1 N0 M0	No

†Goblet cell features. UA, unavailable.

## Discussion

The main finding of our report is that postoperative ^68^Ga‐DOTATATE PET has a low yield in incidentally detected appendiceal NETs and thus, its routine use in this setting may not be indicated. Further, the majority of appendiceal NETs have favourable histopathologic features and clinical outcome. Accordingly, reserving postoperative ^68^Ga DOTATATE imaging for patients with high‐risk features at surgery or histopathology may be more appropriate.

Tumour size is typically considered the most important factor in risk stratification and forms the basis of the ENETS guidelines on diagnostic imaging in these tumours.^2^, ^4^, ^12^ Although there is consensus around imaging and treatment approaches for appendiceal NETs smaller than 10 mm and larger than 20 mm, this is not the case for those between 10 and 20 mm. Our data not only suggest that these subjects tend to have good outcomes, but that postoperative imaging with ^68^Ga‐DOTATATE PET is unnecessary. Avoiding imaging in this group would reduce exposure to ionizing radiation, allay patient anxiety, as well as decrease health care costs.

Table three reinforces the idea that postoperative PET scans are usually negative, even in subjects with a higher grade or more adverse features, histologically. Although we lacked outcome data in a number of patients who underwent right hemicolectomy, we nevertheless feel that tumours with goblet cell features and/or higher Ki67 indices may still require postoperative imaging. In these cases, even though goblet cell tumours can express somatostatin receptors, we believe that ^18^F‐FDG PET would be more suitable than ^68^Ga‐DOTATATE PET.^15^, ^16^, ^17^

There are several potential limitations in our study. First, this is a small cohort and single centre experience, and it is possible that our data may not be generalizable. However, our conclusion regarding the low yield of ^68^Ga‐DOTATATE PET does mirror data from another institution,^18^ as well as recommendations from recent reviews.^19^, ^20^ and appropriate use criteria.^21^ Second, it is possible that the retrospective nature could have introduced bias. For example, the combination of a tertiary hospital experience and specialist referral patterns may have led to an overestimation of the value of imaging in this setting. However, the low yield of postoperative ^68^Ga‐DOTATATE PET in our study contrasts with what might have otherwise been expected and thus, reassures us that the effect of any bias is small.

We conclude that ^68^Ga‐DOTATATE PET is unnecessary for most patients following the diagnosis of an incidental appendiceal NET. Most patients exhibit a good clinical outcome and PET imaging should be reserved for patients exhibiting high‐risk features either at surgery or on histopathology.

## Author contributions

**Emilie Pauvert:** Data curation; formal analysis; investigation; methodology; writing – original draft; writing – review and editing. **George Larcos:** Conceptualization; formal analysis; methodology; project administration; supervision; writing – review and editing.

## Conflicts of interest

None declared.
